# Supplementary material for: The Identification of Nuclear FMRP Isoform Iso6 Partners
Source: Cells. 2023 Dec 9;12(24):2807. doi: 10.3390/cells12242807 (PMC10742089; doi:10.3390/cells12242807)
Supplement: Supplementary file 1 [file cells-12-02807-s001.zip › Supplementary figures S1-S3.pdf]

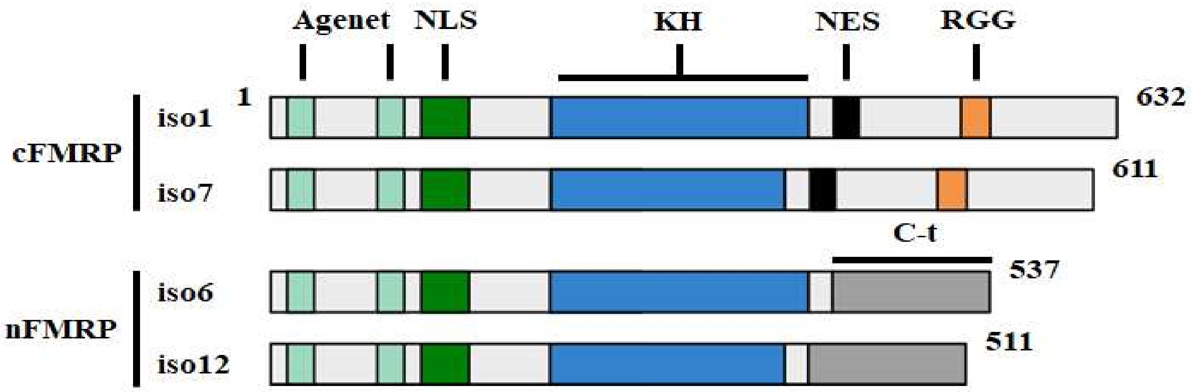

Supplementary figure S1. A schematic representation of the major cytoplasmic (iso1/7) and nuclear (iso6/12) isoforms, as noted in NCBI. The common functional domains and the specific C-t domain of iso6/12 are indicated.

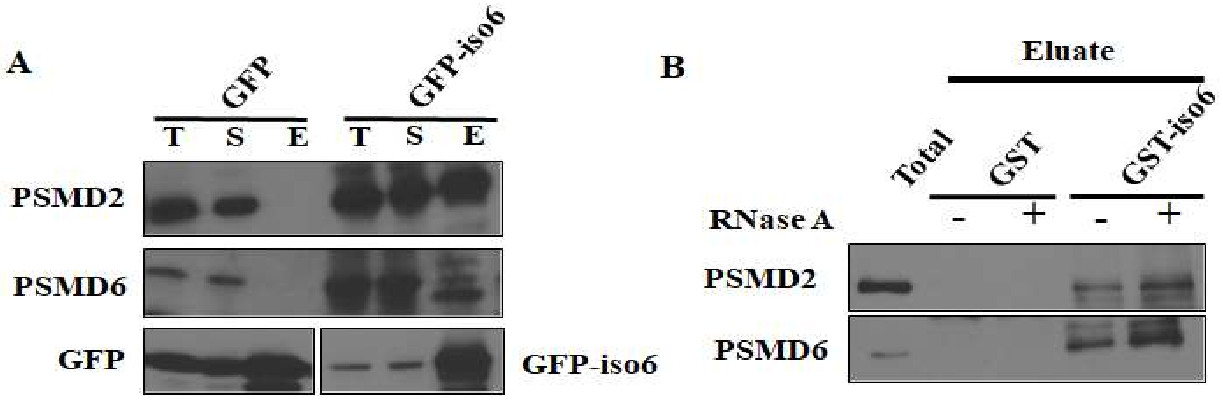

Supplementary figure S2. (A) U2OS-stably expressing either GFP-iso6 or a GFP control were lysed and their proteins pulled-down in GFP-trap as in figure 1. Bound PSMD2, PSMD6 and PSMC5 proteins were analysed by western blot analysis using specific antibodies. T: total extract. S: unbound proteins. E: eluates. (B) GST-pull down as described in figure 1E, with or without RNase A treatment. Bound proteins were analysed by western blot analysis using specific antibodies.

A

Functions	Genes
RNA modification, RNA structure and stability	FXR1<br>DDX41<br>RBM14<br>PRPF31<br>POLDIP3<br>ELAVLs<br>RBM15B<br>ZFR<br>RCL1<br>HNRNPAs<br>RTCB<br>ADAR<br>ZC3H11A
Transcription process, RNA synthesis	TCF20<br>SCRT1<br>CDK9<br>YEATS4<br>THOC5<br>TBL1XR1<br>GATAD2s<br>DPF2<br>GTF2I<br>ADNP<br>BCL11B
DNA binding	ZNF512B<br>ZNF638

Functions	Genes
Microtubules metabolism	ATAT1<br>DCX
Nuclear pore complex	NUPs<br>POM121
Ribosome biogenesis and maturation	WDR33<br>UTP14A
Mitochondrial metabolism	AUH
Chromatin remodeling and chromosome condensation and segregation, Nucleosome remodeling	PBRM1<br>EP400<br>SMC1A<br>SMCHD1<br>CHD4
DNA replication and DNA repair DNA damage response	LIG3<br>PPP1R10<br>WIZ<br>TAOK2<br>SHOC2<br>RBBP6
Mitose regulation	SEPTIN9

B

Functions	Genes
RNA modification, RNA structure and stability	FXR1<br>POLDIP3<br>ELAVL1<br>ADAR<br>ZC3H11A
Transcription process, RNA synthesis	GATAD2B<br>ADNP
DNA replication and DNA repair DNA damage response	PPP1R10
Nuclear pore complex	NUP93
Ribosome biogenesis and maturation	UTP14A
Chromatin remodeling and chromosome condensation and segregation, Nucleosome remodeling	CHD4

Supplementary figure S3. Comparative analysis of proteins binding to FMRP between our study and the Kieffer et al (2022) study conducted in rat brain tissue. (**A**) Interactions identified by *in vitro* GST pull-down/mass spectrometry analysis with the N-terminal common part of FMRP on a nuclear fraction isolated from rat forebrains, (Kieffer et al 2022). (**B**) Interactions identified in both Kieffer et al study and in our GFP-trap/mass spectrometry analysis of U2OS proteomes using GFP-iso6 as a bait (Supplementary table 1).
